# Feedback training induces a bias for detecting happiness or fear in facial expressions that generalises to a novel task

**DOI:** 10.1016/j.psychres.2015.11.007

**Published:** 2015-12-30

**Authors:** Sarah Griffiths, Chris Jarrold, Ian S. Penton-Voak, Marcus R. Munafò

**Affiliations:** a School of Experimental Psychology, University of Bristol, Bristol, United Kingdom; b Medical Research Council Integrative Epidemiology Unit (MRC IEU) at the University of Bristol, Bristol, United Kingdom; c UK Centre for Tobacco and Alcohol Studies, University of Bristol, Bristol, United Kingdom

**Keywords:** Emotion, Intervention, Perception, Sensitivity, Bias

## Abstract

Many psychological disorders are characterised by insensitivities or biases in the processing of subtle facial expressions of emotion. Training using expression morph sequences which vary the intensity of expressions may be able to address such deficits. In the current study participants were shown expressions from either happy or fearful intensity morph sequences, and trained to detect the target emotion (e.g., happy in the happy sequence) as being present in low intensity expressions. Training transfer was tested using a six alternative forced choice emotion labelling task with varying intensity expressions, which participants completed before and after training. Training increased false alarms for the target emotion in the transfer task. Hit rate for the target emotion did not increase once adjustment was made for the increase in false alarms. This suggests that training causes a bias for detecting the target emotion which generalises outside of the training task. However it does not increase accuracy for detecting the target emotion. The results are discussed in terms of the training’s utility in addressing different types of emotion processing deficits in psychological disorders.

## Highlights

• Participants were trained to detect happiness or fear in low intensity expressions.
• Training lead to a bias for recognizing happiness or fear on faces in a novel task.
• However, participants did not become more accurate at recognizing happiness or fear.
• This training may be effective in altering specific biases in emotion recognition.

## 1 Introduction

Many psychological disorders have been associated with differences in the interpretation of emotional facial expressions. These may be the result of differences in response criterion for certain emotions (biases) and/or the result of differences in perceptual sensitivity (Yoon et al., 2014). Reduced perceptual sensitivity for certain emotional expressions has been found in autism spectrum disorder (Law Smith et al., 2010, Wallace et al., 2011), anxiety disorders (Frenkel et al., 2009) and eating disorders (Ridout et al., 2012). Whereas bias for perceiving sadness has been found in depression (Bourke et al., 2010) and anxiety disorder (Bell et al., 2011), while bias for perceiving anger has been found in conduct disorder (Schönenberg and Jusyte, 2014) and social anxiety (Yoon et al., 2014). Differences in bias and sensitivity to certain emotions are commonly measured using low intensity facial expressions, as insensitivity and bias may only be evident when cue intensity is reduced (Frenkel et al., 2009, Yoon et al., 2014).

Although general emotion recognition training programmes have been developed for individuals with various psychological disorders (Wölwer et al., 2005, Ryan and Ni Charragain, 2010, Dadds et al., 2012), there have been few attempts to deliver training which targets emotion specific deficits. Given findings of selective impairments in sensitivity to particular emotional expressions (Frenkel et al., 2009, Law Smith et al., 2010, Ridout et al., 2012), targeted training to improve sensitivity to one specific expression may be an effective treatment. One way of doing this may be to focus training on encouraging recognition of low intensity expressions of a particular emotion.

A number of procedures have been developed to address cognitive biases in psychiatric populations. These commonly aim either to modify *attentional* biases by training attention away from particular emotional stimuli (Heeren et al., 2015), or to modify *interpretation* biases by training participants to interpret ambiguous scenarios as emotionally positive. More recently a paradigm has been developed which aims specifically to modify biases in the perception of ambiguous facial expressions (Penton-Voak et al., 2012, Penton-Voak et al., 2013). In this training, participants are presented, in a random order, with facial expressions from a 15-step morph sequence between a happy and angry (or sad) expression. They are asked to categorise each expression as being one of the two emotions. The point in the morph sequence at which each participant’ switches from one emotion category to another is determined at baseline. In training participants categorise the expressions again, this time receiving feedback about their accuracy after each response. Participants in the active training group receive feedback telling them that 2 more expressions just on the angry (or sad) side of their own category boundary (i.e. relatively ambiguous expressions that they characterized, on average as angry at baseline) are actually happy. Participants in the control group receive feedback that is consistent with their baseline category boundary. Penton-Voak et al. (2013) trained youths at risk of criminal offending to categorise more expressions in a happy–angry morph sequence as happy. Immediately after training and two weeks later, those who received training to modify their category boundary showed reduced self-reported and observer-reported aggressive behaviour, compared to those who received control training. This suggested that training which targets the interpretation of low intensity expressions can affect interpretation of emotions outside the lab and have a positive effect on mood and behaviour.

It is assumed that the effects on mood and behaviour observed in these facial expression recognition training studies are the result of altered perception of facial expressions that are encountered in social interactions. However, this was not explicitly tested by Penton-Voak et al. (2013), and it is not clear exactly how this form of training is influencing perception. Penton-Voak et al. (2013) showed that a larger number of expressions were being identified as happy in the training task, but did not test perception of other emotional expressions or perception of expression on other faces. It is unclear whether training increased sensitivity to happiness or induced a bias to perceive all ambiguous expressions positively. As some psychological disorders are associated with biases towards negative emotions, inducing a bias towards positive emotions has potential therapeutic value. However, if morph sequence feedback training is to address deficits in sensitivity, it must increase discrimination accuracy. It is therefore important to know whether or not morph sequence feedback can increase accuracy in order to decide how this form of training may be best applied.

In this study we tested whether morph sequence feedback training can increase recognition accuracy for a particular emotion. The training method was based on the procedure described by Penton-Voak et al., 2012, Penton-Voak et al., 2013). However, instead of using stimuli from morph sequences that morphed between two emotional expressions, we morphed from one expression, through an ambiguous expression (created by averaging 7 expressions) and towards the corresponding anti-expression (an expression in which the features have moved in the opposite direction from the emotional expression e.g., eyebrows are raised rather than lowered). We chose to morph the emotional expressions with an ambiguous expression, rather than another emotional expression, because we wanted to increase the chances of improving sensitivity to one particular emotion without reducing sensitivity to another. As the anti-expressions at the end of the sequence do not correspond strongly to a particular emotional state, reducing sensitivity to these anti-expressions should not impair emotion recognition ability.

Participants were either trained to recognise happiness using a happy to anti-happy sequence or a fear using a fear to anti-fear sequence. Happiness was chosen because it has been the focus of previous training studies (Penton-Voak et al., 2012, Penton-Voak et al., 2013) and poor recognition of happiness has been associated with depression (Surguladze et al., 2004). Fear was chosen because particularly poor recognition of fear has been found in conditions such as conduct disorder (Marsh and Blair, 2008). Participants categorised faces from the morph sequences by whether or not they showed the target emotion. A 6 alternative forced choice emotion labelling task, which included 15 levels of expression intensity, was completed before and after training. In order to determine how training altered sensitivity and bias for recognition of the target expression, change in hit rate, number of false alarms, and unbiased hit rate (Wagner, 1993) for happy and fearful expressions were compared between training groups. It was predicted that modification feedback training would alter recognition performance for the target emotion (either happiness or fear depending on training group) in the labelling task, while control feedback training would not.

## 2 Method

### 2.1 Participants

One hundred and twenty participants were recruited from a database of volunteers at the University of Bristol. One participant withdrew during the training session, leaving data from 119 participants. All had normal or corrected to normal vision. Participants received 5 GBP or course credit for their participation. Each participant was randomly assigned to one of four training conditions; happy modification (*N*=29, 19 female, mean age=21, *SD*=3.09), happy control (*N*=30, 24 female, mean age=20, *SD*=1.65), fear modification (*N*=31, 26 female, mean age=21, *SD*=2.14), or fear control (*N*=29, 20 female, mean age=21, *SD*=3.67).

### 2.2 Design

The experiment used a 2×2 between-subject design. The factors were target emotion in training (fear or happy) and feedback type in training (modification or control). Manipulating feedback type allowed us to determine whether any effects were the result of feedback, or simply exposure to the target emotion during the training procedure. Manipulating target emotion allowed us to determine whether the training had consistent effects when applied to different emotions.

In the training task the dependent variable was change in “threshold”; the proportion of morph sequence steps participants categorised as the target emotion. In the forced choice labelling task, the dependent variables were change in hit rate, change in number of false alarms and change in unbiased hit rate for the target emotions from before to after training. All participants labelled happy and fear among 4 other emotions in the labelling task. Increases in false alarms for the target emotion (detecting the target emotion in non-target expression) in a particular training group would indicate that that group had developed a bias for detecting that emotion. Participant groups were compared on their responses for the two target emotions in order to determine whether any training effects were emotion-specific or -general (e.g., does training on fear make only fear recognition more accurate, or does it also make recognition of happy more accurate). Therefore, there was an additional within-subject factor of “tested emotion” (happy, fear) in the forced choice task.

Sample size was determined from a power calculation based on previous morph sequence training studies. The effect size for the change in threshold in training previous studies was *d*~1.0 (Penton-Voak et al., 2012, Penton-Voak et al., 2013). The power calculation suggested that 23 participants would be needed in each group to achieve 95% power to detect this change with an *α* level of 0.05. We chose to recruit 30 participants to each group, as effects on the forced choice transfer task were likely to be smaller than effects in the training task.

### 2.3 Materials

**Fig. 1:**
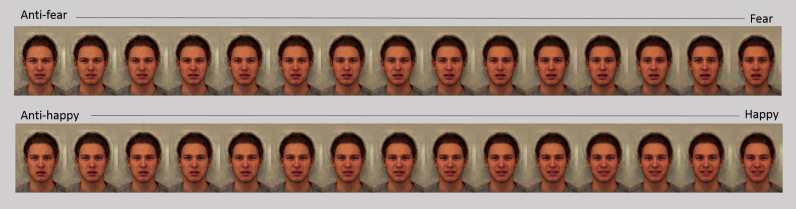
Anti-happy to happy and anti-fear to fear morph sequences pictures used in the training task.

Training task stimuli were pictures of 15 expressions from a happy to anti-happy morph sequence and 15 expressions from a fear to anti-fear morph sequence displayed on a single composite male face. The composite face was created by averaging photographs of 20 adult males taken from the Karolinska Directed Emotional Faces (Lundqvist et al., 1998). The computer programme Psychomorph was used to average shape, colour and texture information across pictures of the 20 faces posing each of the 6 basic facial expressions (happy, sad, angry, surprised, disgusted and fearful) (Tiddeman et al., 2001). The morph sequence was created by morphing the happy and fearful composite faces with an emotionally ambiguous composite face. The emotionally ambiguous face was created by averaging all pictures of the 20 males showing the 6 expressions plus a neutral expression. The morph sequences between each expression and the ambiguous expression were then extended past the ambiguous expression to create anti-expressions (Skinner and Benton, 2010). See Supplementary Information for further details of this process. 15 equally spaced steps between the 100% intensity expression and the 30% anti-expression were then selected (see Fig. 1). Therefore, moving along the sequences, the first 3 pictures contained components of the anti-expressions, while the next 12 pictures contained components of the veridical expression at increasing intensity levels. Including the 30% anti-expression at the end of the sequence, rather than stopping at the ambiguous expression ensured that roughly half the morph sequence pictures were categorised as the target emotion at baseline. This was to keep the training consistent with that employed in previous studies (Penton-Voak et al., 2012, Penton-Voak et al., 2013) to stop a bias developing simply because of the predominance of one response at the outset.

**Fig. 2:**
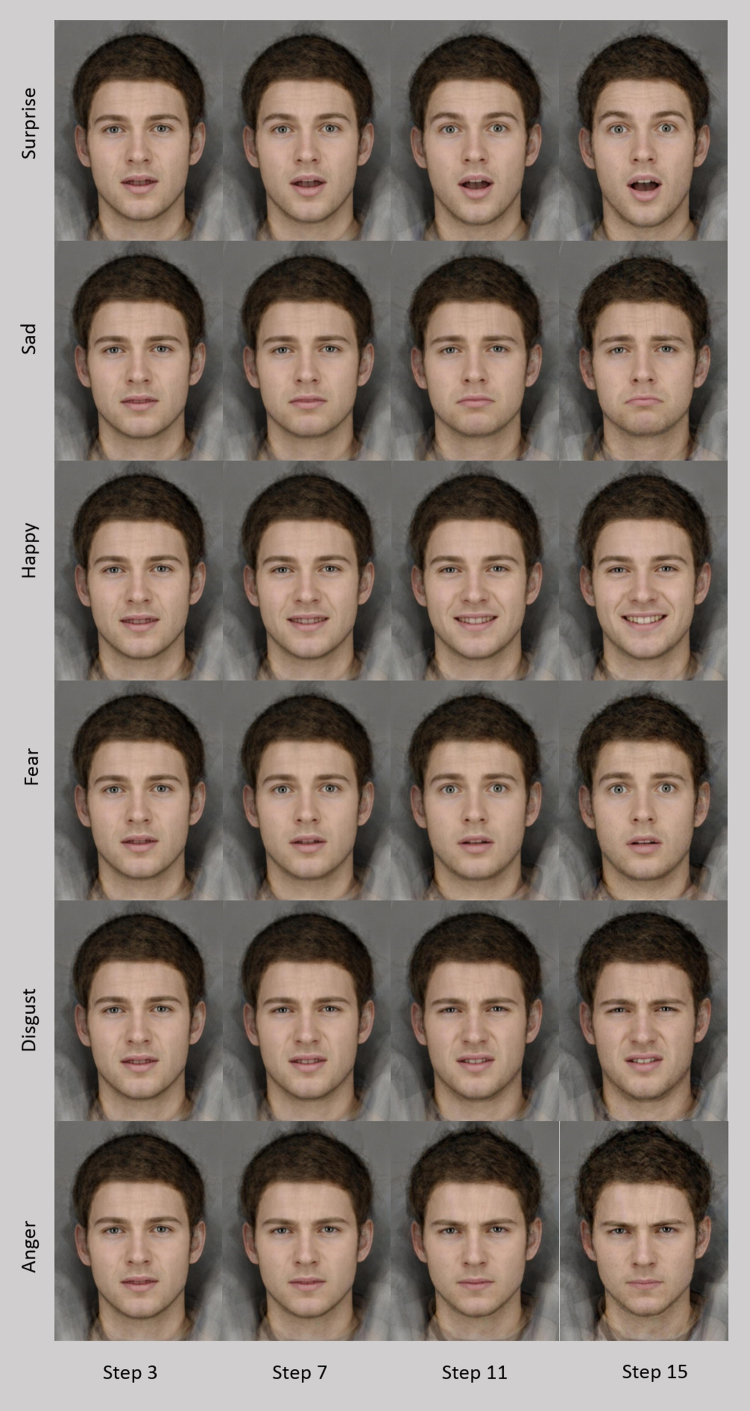
Selected pictures from forced choice task.

To ensure any training effects were transferable across facial identities, a different composite face was created for use in the forced choice task. This was created from photos of 12 adult males posing each expression, taken in our laboratory at the University of Bristol. An emotionally ambiguous expression was created for this face in the same way as described above. Each of the 6 emotional expressions were morphed with the ambiguous expression and 15 equally spaced steps were selected from each sequence (see Fig. 2). These sequences were not extended to include the anti-expression so each step in the sequences showed increasing intensity levels of the expression. This created 6 sequences with 15 steps, giving a total of 90 stimuli.

### 2.4 Procedure

In the forced choice task, participants were asked to decide whether faces presented were happy, sad, angry, fearful, surprised or disgusted. In each trial participants saw a fixation cross appear in the centre of the computer screen (screen dimensions 30×48 cm^2^, viewing distance 50 cm) for between 1500 and 2500 ms (randomly jittered), followed by an picture of a face for 150 ms, followed by a mask of visual noise for 250 ms. The 6 emotion labels then appeared the screen in a circular formation. The positions of the labels were randomly selected for each participant and stayed the same throughout the testing session. The labels stayed on the screen until the participant had responded by selecting a label with the mouse, at which point the next trial started. There were two blocks of 90 trials (180 trials in total) in which each stimulus was presented once in each block (twice in total), with a break in the between blocks. Presentation order was random within blocks. Participants completed this task twice, once before and once after the training procedure.

The training procedure consisted of three phases: baseline, feedback and test. In all phases participants judged whether or not faces from the happy (or fearful) morph sequence showed a happy (or fearful) expression. In each phase, participants saw the 15 pictures from the morph sequence presented in a random order. In the baseline and test phases each picture was shown 3 times, giving a total of 45 trials. In the feedback phase there were 6 blocks of 31 trials in which pictures 1–2 and 14–15 were presented once, pictures 3–5 and 11–13 were presented twice and pictures 6–10 were presented three times. This was to focus training on the critical steps in the morph sequence around the likely threshold. The proportion of faces each participant judged to be happy or fearful at baseline was used to select the baseline threshold for recognising that emotion, corresponding to a particular step in the morph sequence. In the feedback phase, participants in the control condition received feedback consistent with their baseline threshold, whereas participants in the modification condition received feedback that attempted to shift their baseline threshold by two steps, so an extra two steps on the non-emotional side of the boundary were categorised as happy/fearful. This 2-step change is the same as in previous training (Penton-Voak et al., 2012, Penton-Voak et al., 2013). In each trial, participants were presented with a central fixation cross for 1500–2500 ms (randomly jittered), followed by a face (562 by 762 pixels) for 150 ms. A mask of visual noise was then presented for 150 ms, after which a central question mark was displayed until participants responded by pressing one of two keys on the keyboard. In the training blocks, responses were followed by a message, displayed for 1000 ms, saying ‘Correct/Incorrect! That face was happy/fearful/not happy/not fearful’

### 2.5 Analysis

#### 2.5.1 Training task

Change in morph sequence thresholds from baseline to test phases was analysed to check that modification feedback was increasing the tendency to detect the target emotion. Change in threshold was entered in to a 2×2 between subjects ANOVA with target emotion (fear, happy) and feedback type (modification, control) as factors. One sample *t*-tests were then carried out to determine whether any group showed a change in threshold that was different from zero.

#### 2.5.2 Forced choice emotion labelling task

Hit rate, number of false alarms and unbiased hit rate (Wagner, 1993) were analysed. These measures were chosen because we were interested in whether modification training increased target emotion detection rate, and whether this was due to an increase in bias or an increase in sensitivity. Hit rate is the proportion of trials in which a particular emotion is shown that is correctly labelled. Number of false alarms is the number of times in which a particular emotion label is incorrectly used. Unbiased hit rate (*Hu*) gives a measure of perceptual sensitivity by taking in to account both hit rate and false alarm rate. The measure was devised for use in category judgement experiments where other methods of accounting for bias (e.g., signal detection) are inappropriate due to the study design (Wagner, 1993) *Hu* is calculated as: *Hu*=(*Ai*/*Bi*)×(*Ai*/*Ci*), where *Ai*=frequency of hits, *Bi*=number of trials where *i* is target and *Ci*=frequency of *i* responses (hits and false alarms). *Hu* values were arcsine transformed before analysis as suggested by Wagner (1993). The calculation gives hit rate adjusted for the tendency to give that particular response in any trial.

For each of the three variables, change scores were calculated by taking the group mean before training from the mean after training (positive scores therefore indicate improvement). Initially, all outcome measures were analysed in mixed 2×2×2 ANOVAs with tested emotion (happy, fear) as a within-subject factor, and feedback type (modification, control) and target emotion in training (happy, fear) as between-subjects factors. Trials in which anger, sadness, surprise and disgust were shown were only included in the design in order to make the discrimination task sufficiently challenging. We did not include these emotions in our analysis as we did not hypothesise that training would affect performance on these emotions. However, data on performance for the non-target emotions can be found in the Supplementary Information.

Evidence of 3-way interactions in these analyses would suggest that modification training may be having the expected, specific effect on performance on the target emotion. For example, if there was a unique effect of fear modification training on fear recognition an interaction would be seen. Therefore, in cases where this critical 3-way interaction was observed, we carried out separate 2×2 ANOVAs for each target emotion condition, followed up with *t*-tests to make critical comparisons between modification and control groups on performance of the target emotion in training. For brevity, only critical interaction results from the ANOVAs are reported. Full results from all of these analyses can be found in Supplementary information.

The data that form the basis of the results presented here are available to researchers on request from the data.bris Research Data Repository http://dx.doi.org/10.5523/bris.1vx65vv968imz1p8rvhte5vk9a.

## 3 Results

### 3.1 Training task

**Fig. 3:**
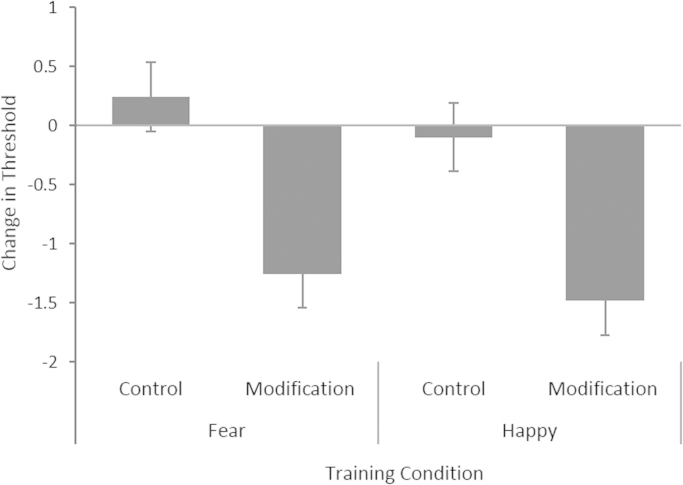
Mean threshold change in training task from baseline to post training for participants in the 4 training conditions. Lower thresholds reflect greater proportions of morph sequence steps being categorised as being the target emotion. Error bars show standard error.

The 2×2 ANOVA revealed evidence for a main effect of feedback type (*F* [1, 115]=24.722, *p*<0.001, *η*^2^=0.177) but no evidence for a main effect of target emotion (*F* (1,115)=0.954, *p*=0.331, *η*^2^=0.008), and no evidence for an interaction (*F* (1,115)=0.041, *p*=0.841, *η*^2^<.001). One sample *t*-tests provided evidence for decrease in threshold (the proportion of morph sequence steps participants categorised as the target emotion) from pre- to post-training in the groups who received modification feedback (fear, *t*(30)=−5.130, *p*<0.001; happy, *t*(28)=−3.936, *p*<0.001) but no evidence for a change threshold in groups who received control feedback (fear, *t*(28)=0.754 *p*=0.457; happy, *t*(29)=−0.516, *p*=0.610) (see Fig. 3.)

### 3.2 Forced choice task emotion labelling task

#### 3.2.1 Hit rate

**Fig. 4:**
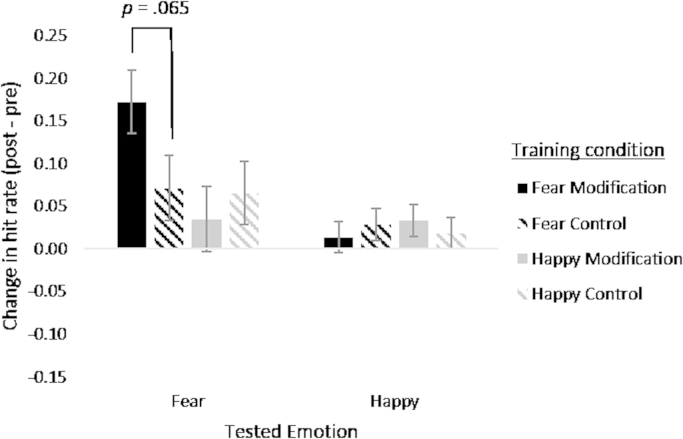
Mean change in hit rate for happy and afraid faces in the forced choice task for the participants in the 4 training conditions. Error bars show standard error.

The 3-way ANOVA indicated evidence for the critical interaction between tested emotion, feedback type, and target emotion (*F* [1, 115]=4.15, *p*=0.044, *ɳ*^2^=0.035). Separate 2-way ANOVAs on the two target emotion conditions indicated evidence for an interaction between feedback type and tested emotion in the fear target training group (*F* [1, 56]=4.61, *p*=0.036, *ɳ*^2^=0.074), but not in the happy training group (*F* [1, 57]=0.61, *p*=0.44, *ɳ*^2^=0.011). This reflects the fact that participants who received fear modification feedback showed a greater increase in fear hit rate than those who received fear control feedback (post-hoc *t*-tests provided weak statistical evidence for this difference; *t*(58)=1.88, *p*=0.065). However, participants who received happy modification feedback did not show a greater increase in happy hit rate than those who received happy control feedback (*t*(57)=0.53, *p*=0.60) (Fig. 4). This is likely due to a ceiling effect for happy hit rate as it is nearing 90% in all conditions.

#### 3.2.2 Number of false alarms

**Fig. 5:**
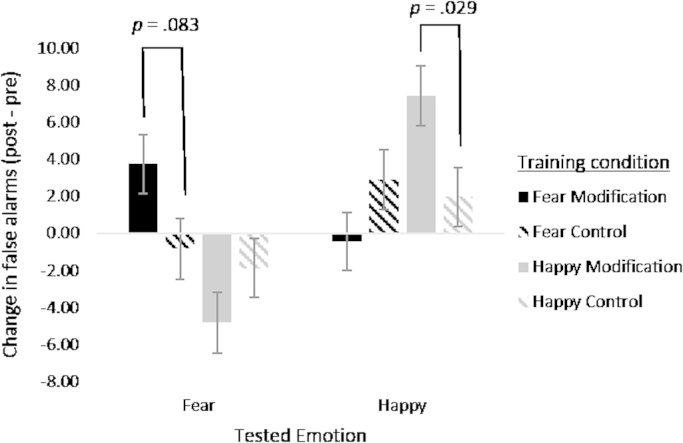
Mean change in number of false alarms for happy and afraid faces in the forced choice task for the participants in the 4 training conditions. Error bars show standard error.

The 3-way ANOVA indicated evidence for the critical interaction between tested emotion, feedback type and target emotion (*F* [1, 115]=10.44, *p*=0.002, *η*^2^=0.083). Separate 2-way ANOVAs on the two target emotion conditions indicated evidence for an interaction between feedback type and tested emotion in both fear (*F* [1, 58]=4.72, *p*=0.034, *ɳ*^2^=0.075) and happy (*F* [1, 58]=5.77, *p*=0.020, *ɳ*^2^=0.092) target conditions. These interactions reflect a greater increase in false alarms for the target emotion after modification feedback compared to control feedback for the happy target condition (*t*(57)=2.24, *p*=0.029) and fear target condition (*t*(58)=1.76, *p*=0.083) (see Fig. 5), although the statistical evidence from post-hoc *t*-tests was weaker for the difference in the fear target condition. The fact that there is a difference in happy false alarms despite no corresponding difference in happy hit rate further suggests a ceiling effect for happy hit rate.

#### 3.2.3 Unbiased hit rate (Hu)

**Fig. 6:**
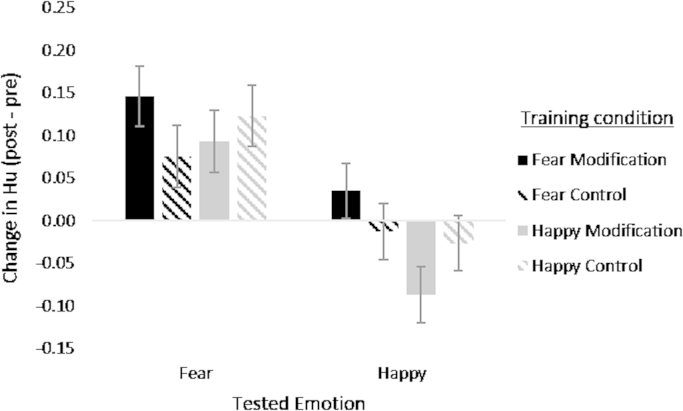
Mean change in unbiased hit rate for happy and afraid faces in the forced choice task for the participants in the 4 training conditions. Error bars show standard error.

The 3-way ANOVA did not indicate evidence for the critical 3-way interaction between tested emotion, feedback type and target emotion (*F* [1, 115]=0.007, *p*=0.934, *ɳ*^2^<0.001), suggesting that there is no effect of specific training condition on sensitivity to the target emotion (Fig. 6). There was a main effect of tested emotion, which reflected overall increase in unbiased hit rate for fear but not for happiness. As this increase is not related to training condition, it is likely a practice effect for recognising fear in the labelling task. The lack of practice effect for recognition of happiness may be due to ceiling effects. Additionally there was some evidence for an interaction between target emotion and feedback type (*F* [1, 115]=3.894, *p*=0.051, *ɳ*^2^=0.033). This reflects the fact that the fear modification group showed a greater increase in overall performance than the happy modification group (fear modification *M*=0.181 *SD*=0.314; happy modification *M*=006 *SD*=0.239, *t*(58)=2.42, *p*=0.019) while the fear control and happy control groups did not differ in overall improvement (fear control, *M*=0.063, *SD*=0.283; happy control *M*=0.097, *SD*=0.307, *t*(57)=0.43, *p*=0.669). However, given there is no evidence that the fear modification group showed a greater overall improvement than the fear control group (*t*(58)=1.52, *p*=0.133) it cannot be concluded that fear modification training improves performance.

## 4 Discussion

We found evidence that morph sequence feedback training targeting happy or fearful expressions, influences the interpretation of other emotional expressions encountered in a transfer task. In the transfer task, which involved labelling 6 different emotional expressions at varying intensity levels, participants who received modification training showed an increase in false detections of the target emotion, indicating training had caused an interpretation bias. Participants who received fear modification training also showed an increase in correct detections of fear, although those who received happy modification training did not show an increase in correct detections of happiness. Critically however, there was no evidence for an increase in sensitivity for the target emotion after either happy or fear modification training.

Previous studies using feedback training to increase the number of expressions in happy–sad and happy–angry morph sequences that are categorised as happy, found decreases in negative affect (Penton-Voak et al., 2012) and decreases in self- and observer-reported aggression after modification feedback (Penton-Voak et al., 2013). It was suggested that the mechanism by which this training is affecting mood and behaviour is through inducing a bias for perceiving happiness in facial expressions encountered outside of the training paradigm. This study adds support to this claim by showing that it is possible for morph sequence feedback training to induce a bias for a certain expression in an emotion recognition transfer task.

There were slight differences in our morph sequence training method compared to the morph sequence training methods used in previous studies. First, we used morph sequences which ran from one expression to it’s corresponding anti-expression, rather than between two emotional expressions. Second, participants decided whether the emotion was present or not, (e.g., “happy or not happy”) rather than deciding which of two expressions were present (“happy or sad”). These changes were made to ensure that we did not train people *not* to select one particular emotion. However, there is no reason why either of these changes would make it more likely that we would find a bias that transferred to another task following training. We are therefore reasonably confident that our findings are applicable to the morph sequence training technique used in previous studies.

Our results suggest that morph sequence training causes a bias towards choosing a particular emotional category. The fact that this bias transferred to another task, which used different face stimuli, required a different decision (six options instead of two) and a required a different motor response (mouse click instead of button press) suggests the training effect is not limited to a simple task-related response bias. However, it is not clear from the current results whether this bias is specific to recognition of emotions from facial expression, or a general bias for all decisions about emotional stimuli. It would be possible to distinguish between these two possibilities by testing the effects of training on the classification of emotional stimuli other than facial expressions (e.g., postures or tones of voice).

Training procedures used in previous studies have aimed to increase the detection of happiness (Penton-Voak et al., 2012, Penton-Voak et al., 2013). In this study we looked at both happiness and fear training and found that both increased false alarms for the targeted emotion, although only fear training increased target hit rate. This is likely due to the happy hit rate being at ceiling in the control training conditions. Ceiling performance for happy expressions in the emotion labelling task is perhaps not surprising as happiness has been shown to be the easiest emotion to identify (Calvo and Lundqvist, 2008, Tottenham et al., 2009). The fact that training effects were evident for training on both emotions suggests this procedure could be used to induce biases for detecting any emotion of interest in future studies.

It is notable that perceptual sensitivity did not improve following training. We had hypothesised that training using morph sequences which included low intensity examples of a particular expression would increase perceptual sensitivity to that expression; but we found this not to be the case. However, the participants in our study were adults with no known deficits in emotion recognition. Given that healthy adults are typically good at detecting basic facial expressions, it may be that improving sensitivity beyond baseline was not possible (Blanch-Hartigan et al., 2012). Increases in sensitivity may be more likely in populations with particular deficits in recognising the target emotion. Nonetheless, the current results suggest that this type of morph sequence feedback training may not be useful in addressing reductions in sensitivity to emotional expressions found in conditions such as autism spectrum disorders (Wallace et al., 2011). However, it may hold promise for addressing biases in facial expression perception, such as those found in depression (Bourke et al., 2010), anxiety disorders (Bell et al., 2011, Yoon et al., 2014) or conduct disorders (Schönenberg and Jusyte, 2014).

Future studies should focus on determining whether alternative training techniques can improve sensitivity to low intensity expressions, as this seems to be a particular problem in many disorders (Frenkel et al., 2009, Law Smith et al., 2010, Ridout et al., 2012). One promising method of training sensitivity using low intensity facial expression has been published recently (Schonenberg et al., 2014). In this study, violent offenders showed increased sensitivity for low intensity expressions after they were implicitly trained to attend to low intensity fearful faces rather than neutral faces using a dot probe paradigm. One possible reason why this training was more effective than that used in the current study is that the expressions were displayed side by side which may have helped to demonstrate the difference between expressions which do and do not show the target emotion.

In conclusion, the current study adds to the findings of previous studies showing that feedback training using expression morph sequences can lead to an increased tendency to detect a particular target emotion (Penton-Voak et al., 2012, Penton-Voak et al., 2013). Our findings show that it is possible to increase the tendency to detect fear as well as happiness. We also demonstrate that the training leads to a bias for detecting the target emotion in any expression rather than an increase in the ability to accurately identify the target emotion expression. Finally, we demonstrate for the first time that this experimentally induced bias in emotion recognition generalises to different identity faces shown in other tasks, suggesting it may have an effect on emotion recognition in faces outside of the lab.
