# Supplementary material for: Feedback training induces a bias for detecting happiness or fear in facial expressions that generalises to a novel task
Source: Psychiatry Res. 2015 Dec 30;230(3):951–7. doi: 10.1016/j.psychres.2015.11.007 (PMC4693450; doi:10.1016/j.psychres.2015.11.007)
Supplement: Supplementary file 1 — Supplementary material [file mmc1.docx]

**SUPPLEMENTARY MATERIAL**

**Stimulus Creation**

All stimuli were created using Psychomorph which can be downloaded for free from: http://users.aber.ac.uk/bpt/jpsychomorph/

Anti-expressions are created by applying a transformation using Psychomorph software. Psychomorph performs transformations using the vectors of 172 delineation points shown on the images below (green crosses). To create anti-expressions the programme calculates the difference between the vectors of for the expression averages (left) and the ambiguous average (middle). 30% of the vector difference is then applied to the ambiguous expressions to transform them into the anti-expressions (right).

Figure S1. Stimuli from the training task with Psychomorph delineation templates shown.


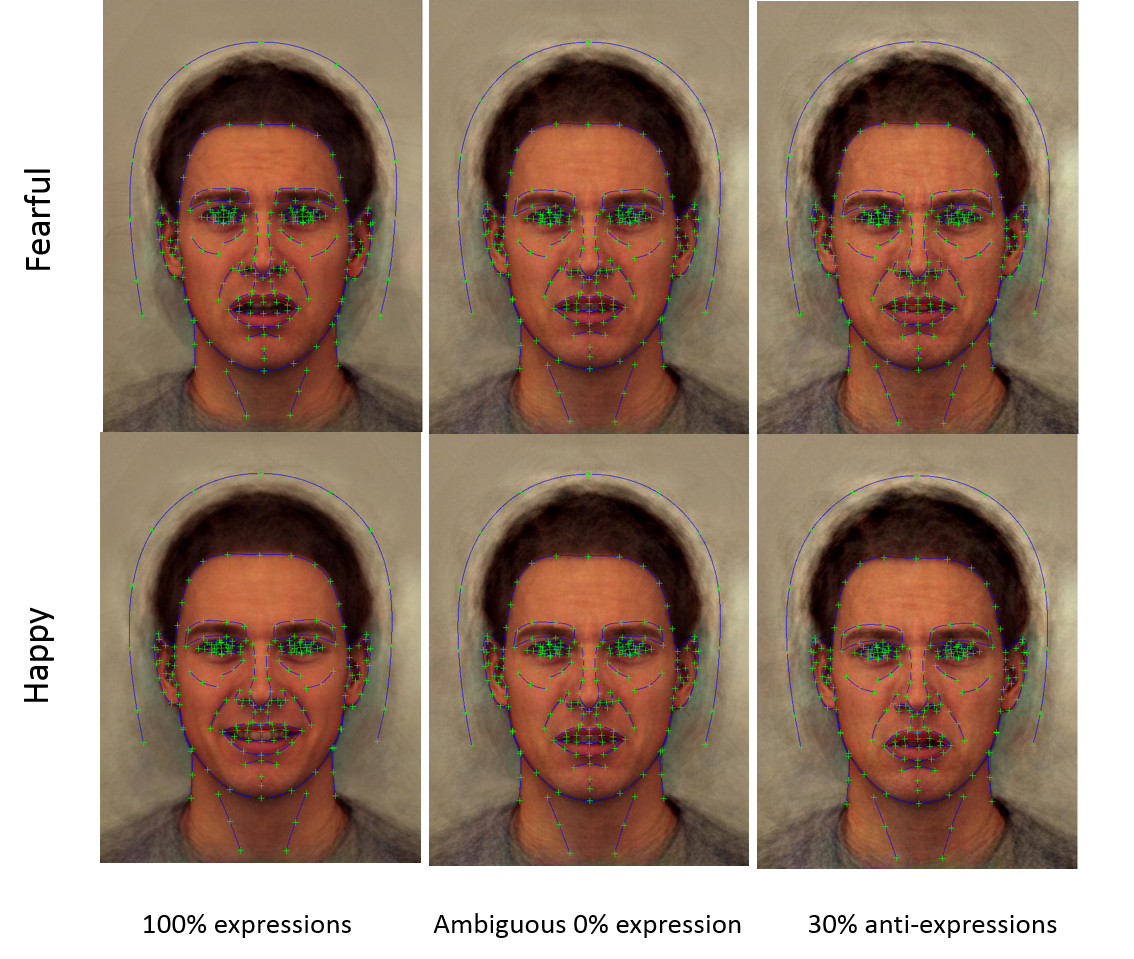


**ANOVA tables from main analysis**

The following tables show the full ANOVA results from the analyses reported in the manuscript.

**Hit rate**

Table S1. 3-way ANOVA of hit rate with tested emotion (happy, fear), feedback type (modification, control) and target emotion in training (happy, fear) as factors

|  | *Df* | *F* | *η2* | *P* |
| --- | --- | --- | --- | --- |
| Within Subjects | | | | |
| Tested emotion | 1 | 9.971 | 0.080 | 0.002 |
| Tested emotion x Target emotion | 1 | 3.696 | 0.031 | 0.057 |
| Tested emotion x Feedback type | 1 | 0.791 | 0.007 | 0.376 |
| Tested emotion x Target emotion x Feedback type | 1 | 4.153 | 0.035 | 0.044 |
| Error (Target Emotion) | 115 |  |  |  |
| Between Subjects | | | | |
| Target emotion | 1 | 2.328 | 0.020 | 0.130 |
| Feedback type | 1 | 0.635 | 0.005 | 0.427 |
| Target emotion x Feedback type | 1 | 1.350 | 0.012 | 0.248 |
| Error | 115 |  |  |  |

Table S2. 2-way ANOVA of hit rate for participants in the fear training group with tested emotion (happy, fear), feedback type (modification, control) as factors

|  | *Df* | *F* | *η2* | *P* |
| --- | --- | --- | --- | --- |
| Within Subjects | | | | |
| Tested emotion | 1 | 13.892 | 0.193 | < 0.001 |
| Tested emotion x Feedback type | 1 | 4.612 | 0.074 | 0.036 |
| Error (Target Emotion) | 58 |  |  |  |
| Between Subjects | | | | |
| Feedback type | 1 | 1.350 | 0.030 | 0.185 |
| Error |  |  |  |  |

Table S3. 2-way ANOVA of hit rate for participants in the happy training group with tested emotion (happy, fear), feedback type (modification, control) as factors

|  | *Df* | *F* | *η2* | *P* |
| --- | --- | --- | --- | --- |
| Within Subjects | | | | |
| Tested emotion | 1 | 0.711 | 0.012 | 0.403 |
| Tested emotion x Feedback type | 1 | 0.614 | 0.011 | 0.436 |
| Error (Target Emotion) | 57 |  |  |  |
| Between Subjects | | | | |
| Feedback type | 1 | 0.072 | 0.001 | 0.790 |
| Error |  |  |  |  |

**False alarms**

Table S4. 3-way ANOVA of false alarms with tested emotion (happy, fear), feedback type (modification, control) and target emotion in training (happy, fear) as factors

|  | *Df* | *F* | *η2* | *P* |
| --- | --- | --- | --- | --- |
| Within Subjects | | | | |
| Tested emotion | 1 | 9.613 | 0.077 | 0.002 |
| Tested emotion x Target emotion | 1 | 10.718 | 0.085 | 0.001 |
| Tested emotion x Feedback type | 1 | 0.011 | < 0.001 | 0.918 |
| Tested emotion x Target emotion x Feedback type | 1 | 10.436 | 0.083 | 0.002 |
| Error (Target Emotion) | 115 |  |  |  |
| Between Subjects | | | | |
| Target emotion | 1 | 0.466 | 0.004 | 0.496 |
| Feedback type | 1 | 0.890 | 0.008 | 0.347 |
| Training emotion x Feedback type | 1 | 0.097 | 0.001 | 0.756 |
| Error | 115 |  |  |  |

Table S5. 2-way ANOVA of false alarms for participants in the fear training group with tested emotion (happy, fear), feedback type (modification, control) as factors

|  | *Df* | *F* | *η2* | *P* |
| --- | --- | --- | --- | --- |
| Within Subjects | | | | |
| Tested emotion | 1 | 0.015 | < 0.001 | 0.905 |
| Tested emotion x Feedback type | 1 | 4.723 | 0.075 | 0.034 |
| Error (Target Emotion) | 58 |  |  |  |
| Between Subjects | | | | |
| Feedback type | 1 | 0.177 | 0.003 | 0.676 |
| Error |  |  |  |  |

Table S6. 2-way ANOVA of false alarms for participants in the happy training group with tested emotion (happy, fear), feedback type (modification, control) as factors

|  | *Df* | *F* | *η2* | *P* |
| --- | --- | --- | --- | --- |
| Within Subjects | | | | |
| Tested emotion | 1 | 21.087 | 0.270 | < 0.001 |
| Tested emotion x Feedback type | 1 | 5.769 | 0.092 | 0.020 |
| Error (Target Emotion) | 57 |  |  |  |
| Between Subjects | | | | |
| Feedback type | 1 | 0.910 | 0.016 | 0.344 |
| Error |  |  |  |  |

**Unbiased hit rate (Hu)**

Table S7. 3-way ANOVA of unbiased hit rate with tested emotion (happy, fear), feedback type (modification, control) and target emotion in training (happy, fear) as factors

|  | *Df* | *F* | *η2* | *P* |
| --- | --- | --- | --- | --- |
| Within Subjects | | | | |
| Tested emotion | 1 | 37.055 | 0.244 | < 0.001 |
| Tested emotion x Target emotion | 1 | 2.255 | 0.019 | 0.136 |
| Tested emotion x Feedback type | 1 | 0.386 | 0.003 | 0.535 |
| Tested emotion x Target emotion x Feedback type | 1 | 0.007 | < 0.001 | 0.934 |
| Error (Target Emotion) | 115 |  |  |  |
| Between Subjects | | | | |
| Target emotion | 1 | 1.811 | 0.016 | 0.181 |
| Feedback type | 1 | 0.067 | 0.001 | 0.796 |
| Target emotion x Feedback type | 1 | 3.894 | 0.033 | 0.051 |
| Error | 115 |  |  |  |

**ANOVA tables from analysis repeated to include all 6 emotions**

The following ANOVA tables show results from the main analysis but with all 6 emotions included in the Tested emotion factor.

**Hit rate**

Table S8. 3-way ANOVA of hit rate with tested emotion (anger, disgust, fear, happy, sad, surprise), feedback type (modification, control) and target emotion in training (happy, fear) as factors

|  | *Df* | *F* | *η2* | *P* |
| --- | --- | --- | --- | --- |
| Within Subjects | | | | |
| Tested emotion | 5 | 10.443 | 0.083 | < 0.001 |
| Tested emotion x Target emotion | 5 | 2.300 | 0.020 | 0.044 |
| Tested emotion x Feedback type | 5 | 1.420 | 0.012 | 0.215 |
| Tested emotion x Target emotion x Feedback type | 5 | 1.786 | 0.015 | 0.144 |
| Error (Target Emotion) | 575 |  |  |  |
| Between Subjects | | | | |
| Target emotion | 5 | 1.987 | 0.017 | 0.161 |
| Feedback type | 5 | 0.052 | < 0.001 | 0.820 |
| Target emotion x Feedback type | 5 | 1.331 | 0.011 | 0.251 |
| Error | 115 |  |  |  |

Note that when all 6 emotions are included in the ANOVA, the evidence for the 3 way interaction is reduced. This is due to the decrease in statistical power that results from adding the other 4 emotions.

As there was some evidence for the interaction between tested emotion and target emotion in this analysis, we explored this interaction with post-hoc tests. Independent sample t-tests were performed to compare the change in hit rate for each emotion by participants who experienced each training emotion (collapsed across feedback type). Tests provided some evidence for increased hit rate for fear (*t*(117) = 1.931, *p* = 0.056) and decreased hit rate for surprise (*t*(117) = -1.789, *p* = 0.076) when fear was the target in training, compared to when happy was the target in training (See Figure S2 below). Training emotion made no difference to change in hit rate for any other tested emotion (*ts*(117) < 1.065, *ps* > 0.289).

**False alarms**

Table S9. 3-way ANOVA of false alarms with tested emotion (anger, disgust, fear, happy, sad, surprise), feedback type (modification, control) and target emotion in training (happy, fear) as factors

|  | *Df* | *F* | *η2* | *P* |
| --- | --- | --- | --- | --- |
| Within Subjects | | | | |
| Tested emotion | 5 | 13.470 | 0.105 | < 0.001 |
| Tested emotion x Target emotion | 5 | 4.866 | 0.041 | < 0.001 |
| Tested emotion x Feedback type | 5 | 0.413 | 0.004 | 0.840 |
| Tested emotion x Target emotion x Feedback type | 5 | 3.886 | 0.033 | 0.002 |
| Error (Target Emotion) | 575 |  |  |  |
| Between Subjects | | | | |
| Target emotion | 5 | 3.030 | 0.026 | 0.084 |
| Feedback type | 5 | 0.179 | 0.002 | 0.673 |
| Training emotion x Feedback type | 5 | 0.024 | < 0.001 | 0.878 |
| Error | 115 |  |  |  |

**Unbiased hit rate (Hu)**

Table S10. 3-way ANOVA of unbiased hit rate with tested emotion (anger, disgust, fear, happy, sad, surprise), feedback type (modification, control) and target emotion in training (happy, fear) as factors

|  | *Df* | *F* | *η2* | *P* |
| --- | --- | --- | --- | --- |
| Within Subjects | | | | |
| Tested emotion | 5 | 15.080 | 0.116 | < 0.001 |
| Tested emotion x Target emotion | 5 | 0.761 | 0.007 | 0.578 |
| Tested emotion x Feedback type | 5 | 0.701 | 0.006 | 0.623 |
| Tested emotion x Target emotion x Feedback type | 5 | 1.162 | 0.010 | 0.327 |
| Error (Target Emotion) | 575 |  |  |  |
| Between Subjects | | | | |
| Target emotion | 5 | 2.829 | 0.024 | 0.095 |
| Feedback type | 5 | 0.455 | 0.004 | 0.501 |
| Target emotion x Feedback type | 5 | 2.317 | 0.020 | 0.131 |
| Error | 115 |  |  |  |

**Extended graphs including all 6 tested emotion**

The following figures show the change in performance for all emotions tested in the forced choice transfer task for each training condition. These are extended versions of the figures provided in the main manuscript.

Figure S2. Mean change in hit rate for each emotion in the forced choice task for participants in the 4 training conditions. Error bars show standard error.

Figure S3. Mean change in false alarms for each emotion in the forced choice task for participants in the 4 training conditions. Error bars show standard error.

Figure S4. Mean change in unbiased hit rate for each emotion in the forced choice task for participants in the 4 training conditions. Error bars show standard error.
